# Development of a face-validated conceptual model structure for economic evaluation in phenylketonuria, through expert elicitation

**DOI:** 10.1186/s13023-026-04423-1

**Published:** 2026-05-29

**Authors:** Ania C. Muntau, Rongrong Zhang, Anupam Chakrapani, Takashi Hamazaki, Melissa Lah, Danielle J. Ruebel, Suresh Vijay, Roberto T. Zori, Francois Feillet, Nicola Longo, Hope Northrup, Suzanne Hollander, Thomas O’Connell, Jonathan J. Woolley, Ioannis Tomazos

**Affiliations:** 1 University Children’s Hospital, University Medical Center Hamburg-Eppendorf and German Center of Child and Adolescent Health (DZKJ), Hamburg, Germany; 2 PTC Therapeutics Sweden AB, Askim, Sweden; 3 Great Ormond Street Hospital for Children, London, UK; 4 Department of Pediatrics, Osaka Metropolitan University Graduate School of Medicine, Osaka, 545-8585 Japan; 5 Indiana University School of Medicine, Indianapolis, IN USA; 6 Birmingham Children’s Hospital, Birmingham, UK; 7 Pediatric Genetics and Metabolism, University of Florida, Gainesville, FL USA; 8 Reference Centre for Inborn Errors of Metabolism, Department of Pediatrics, Children’s Hospital of Nancy, Nancy, France; 9 Department of Human Genetics, University of California Los Angeles, Los Angeles, CA USA; 10 Division of Medical Genetics, The University of Texas Health Science Center at Houston, Houston, TX USA; 11 Division of Genetics and Genomics, Boston Children’s Hospital, Boston, MA USA; 12 Medicus Economics, Boston, MA USA; 13 PTC Therapeutics Inc, 500 Warren Corporate Center Drive, Warren, N.J 07059 USA

## Abstract

**Background:**

Clinical complexity and limitations of available evidence make cost-effectiveness analyses (CEAs) in phenylketonuria (PKU) challenging, with no consensus model structure emerging from historical CEAs. This research aimed to elicit perspectives from PKU medical experts regarding approaches for CEA modeling, to inform development of a face-validated model structure.

**Methods:**

In a modified e-Delphi study, clinical experts rated their agreement/disagreement with 14 statements on clinical outcomes, diet, target population, blood phenylalanine (Phe)-level classification, and treatment discontinuation. Separately, a survey of a broader sample of experts elicited perspectives on the prevalence of PKU comorbidities, and biological plausibility of the association of surrogate outcomes with comorbidity risk. Insights informed development of the conceptual structure and parameters for a CEA model.

**Results:**

In the modified e-Delphi study, participants (*N* = 11) achieved consensus (≥ 70% agreement/disagreement) on statements on the associations of blood Phe levels with outcomes, unmet need, mechanisms for the association of PKU with chronic comorbidities, and medical management approaches. In the survey (*N* = 30), ≥ 70% of participants agreed that prevalence of comorbidities is higher in PKU vs. the general population, and that increased natural-protein consumption may reduce comorbidity risk, including for depression, osteoporosis, overweight, anemia, and type two diabetes mellitus. Findings informed development of a CEA model structure comprising five blood Phe level-defined health states (range: ≤120 µmol/L to > 1200 µmol/L), associated with four classes of patient-relevant clinical outcomes in PKU: contemporaneous symptoms/manifestations, intellectual disability, comorbidities, and diet liberalization.

**Conclusions:**

In this research, perspectives were elicited from medical experts in PKU to develop a clinically informed, face-validated conceptual model structure for CEA, and to identify associated parameters. The resulting *de novo* economic model allows for valuation of numerous important patient-relevant outcomes, enabling modeling of the heterogeneous drivers of unmet need in PKU. The structured expert-elicitation approach used in this research, and documentation of the influence of perspectives elicited on model structure and parameters, support the validity of the model for application in economic evaluation of interventions for PKU.

**Supplementary Information:**

The online version contains supplementary material available at 10.1186/s13023-026-04423-1.

## Introduction

Phenylketonuria (PKU) is an inherited metabolic disorder characterized by a deficiency in phenylalanine hydroxylase (PAH), the enzyme responsible for converting phenylalanine into tyrosine, leading to elevated blood phenylalanine (Phe) levels [1]. The resulting accumulation of Phe in the brain can cause neurocognitive and neurobehavioral impairments [2–4]. Moreover, elevated blood Phe levels and/or some of the dietary changes that are part of standard management and therapy, including high amino acid based medical food formulas and higher carbohydrate and fat intake due to the limits on natural protein, are associated with an increased risk of chronic conditions such as obesity, renal disease, and cardiovascular disease [5]. Individuals with PKU require meticulous disease management, involving rigorous lifelong dietary restrictions to maintain low blood Phe levels within recommended treatment guidelines [4, 6]. Adherence to these Phe-restricted diets can be challenging, particularly with advancing age. Consequently, neurological damage associated with poorly controlled blood Phe levels, including impacts on executive function, mental-health/mood disorders, and in some cases on intelligence quotient (IQ) score, may persist [7–10]. 

Sapropterin dihydrochloride and pegvaliase, both therapies which aid in breaking down Phe in the blood, have been the main pharmacological treatment options for PKU, first approved in 2007 and 2018, respectively [11, 12]. Recently, sepiapterin, which enhances PAH enzyme activity, has also received approval from the European Commission and from the United States Food and Drug Administration for the treatment of children and adults with PKU [13, 14]. 

Despite the longstanding availability of pharmacological therapies for PKU, a consensus approach to economic modeling of the condition has yet to emerge. A recent review identified seven previous health technology assessments (HTAs) which evaluated either sapropterin or pegvaliase for reimbursement; a significant lack of consensus on cost-effectiveness analysis (CEA) model structure and methods used was observed (Table S1) [15]. These evaluations, typically incorporating CEA that compared the quality-of-life benefits and costs of different treatment strategies, were submitted to HTA bodies in Australia, Canada, England, Ireland, and Scotland. CEA approaches varied between submissions and included lifetime-horizon Markov models, microsimulations, and 1-year horizon decision trees. Several Markov models used health states focused on variations of “controlled”, “partially controlled”, and “uncontrolled” blood Phe levels, and one included 5 blood-Phe ranges. HTA bodies generally critiqued that the modeling approaches did not accurately represent the impacts and clinical management of the disease [15]. Specific critiques included the failure to capture the long-term impacts of uncontrolled blood Phe levels, misalignment between health state definitions and utility/cost impacts, lack of support for diet liberalization modeling, and the omission of PKU associated comorbidities and caregiving impacts, resulting in under-representation of the overall burden of the condition (Table S1) [4]. 

The complexities of PKU, in particular the multifaceted impacts of its pathophysiology, dietary restrictions, and long-term comorbidities, pose challenges for disease modeling. Moreover, the potential relationships between these aspects of the disease are uncertain. The inherent considerations and challenges in PKU management, coupled with the lifelong implications of the disease, necessitate deliberate modeling approaches that accurately capture the clinical nuances and long-term outcomes of different treatment strategies [4]. However, as with other rare diseases, available evidence to inform the necessary components of a model for PKU is limited and uncertain.

Large scale studies are often not feasible in rare diseases, and, due to their rarity, the natural history of these diseases is not well understood. Therefore, research eliciting perspectives from clinical experts who have direct experience with these patient populations can help address evidence gaps. This aligns with best practice guidance from the Professional Society for Health Economics and Outcomes Research (ISPOR), which recommends that development of a conceptual model for economic evaluation should involve consulting widely with “subject experts and stakeholders to assure that the model represents disease processes appropriately and adequately addresses the decision problem” [16]. Study designs including quantitative surveys and Delphi panels can be used to translate expert experience into points of agreement and consensus, as well as illuminate clinical observations for which published evidence may be lacking. Establishing consensus on a clinically accurate CEA model structure would be valuable for future assessments of treatments for PKU. Guidelines for economic modeling highlight the importance of validation to support a model’s ability to make accurate predictions [17]. In particular, clinical-expert involvement ensures that the model structure aligns with the current understanding of medical science and best available evidence, contributing to the model’s face validity [17]. Expert perspectives can be elicited through structured approaches such as the Delphi method, a systematic approach for evaluating consensus through iterative, group-based method for expert elicitation and stakeholder engagement [18, 19]. 

The aim of this research was to develop a clinically informed, face-validated conceptual model structure for a *de novo* model for CEA of PKU treatments, through the elicitation of clinical expert perspectives. Consensus modeling approaches and parameters were identified among PKU medical experts using a modified e-Delphi study, and perspectives on the prevalence of comorbidities and biological plausibility of surrogate outcomes reducing comorbidity risk were elicited via a clinical-expert survey.

## Methods

### Model development

This research implemented a structured methodology to elicit and synthesize expert perspectives on clinically accurate economic modeling for PKU therapies. Per best practices, the study protocol was developed a priori, in order to reduce risk of bias and to standardize the methodology.

First, a targeted review of sapropterin and pegvaliase HTAs was conducted to characterize challenges with the approaches for CEAs in PKU [15]. Findings from this review informed a scoping survey with clinical experts (*N* = 8) to determine the clinical outcomes of greatest importance to model [20]. 

Subsequently, a modified e-Delphi study (*N* = 11) was used to elicit perspectives among a multidisciplinary group of international experts, to identify clinically-accurate modeling approaches for these outcomes. In a Delphi study, expert participants provide opinions in response to sequential “rounds” of questions, informed by intermediate summaries of the group’s opinions from the previous round, encouraging participants to reassess, alter, and/or develop opinions [20]. In healthcare-oriented research, “modified” Delphi studies following the RAND/UCLA Appropriateness Method are common, involving two rounds with an intermediate review and discussion of anonymized responses [18]. In recent years, e-Delphi studies, generally taking the form of a modified Delphi studies with discussion via an online forum, have been used increasingly [21–23], as clinical-expert perspectives gained from these studies as well as from surveys can inform the development of future CEA models and support their face validity.

Given general agreement in the modified e-Delphi study on biological mechanisms that may explain higher prevalence of chronic comorbidities in PKU, but variation in the specific comorbidities, a separate clinician survey was conducted. Participants were experts in the management of PKU (*N* = 30) from 15 countries. The survey elicited clinical-expert perspectives on (1) the prevalence of comorbidities in PKU (2) the biological plausibility that controlling Phe levels may reduce comorbidity risk and (3) the biological plausibility that increasing natural protein consumption may reduce comorbidity risk, in order to inform the modeling of burden and risk of comorbidities in PKU. The focus on assessing biological plausibility was motivated by recent HTA guidance on modeling outcomes associated with surrogate endpoints (i.e. blood Phe and diet restrictions) [21]. Perspectives elicited during the modified e-Delphi study of key opinion leaders experienced in the management of PKU and clinical-expert survey were synthesized to inform a model structure that could be used for CEAs of PKU treatments.

The e-Delphi study and clinical-expert survey study were reviewed by the WCG Institutional Review Board and received waivers of the ethical review requirements. For both the survey and the Delphi panel, all participants provided informed consent prior to participating in the process.

### Participants

#### Modified e-Delphi study

In the modified e-Delphi study, to account for potential heterogeneity in clinical perspectives on PKU, a purposive sampling method was employed to ensure diversity in expertise and representation of multiple perspectives in PKU care. Participants were selected based on their extensive experience in PKU management, including involvement in clinical studies of therapies for PKU to ensure familiarity with the PKU therapies including emerging treatments, and to encompass a range of medical disciplines.

Methodological guidance for modified Delphi studies suggests a sample size of 9–18 participants for in-person panels [19, 22], which is consistent with the sample size in another modified Delphi study conducted recently in PKU [23]. The purposive sampling approach identified 11 participants from five countries (France [*n* = 1]; Germany [*n* = 1]; Japan [*n* = 1]; United Kingdom [UK, *n* = 2]; United States [US, *n* = 6]) with experience in the medical or nutritional management of individuals with PKU. Clinical experience ranged from six to over 20 years; specifically, one participant had 6–10 years of experience, three had 11–20 years, and seven had more than 20 years of experience. Types of experience spanned a wide array of disciplines including pediatrics, metabolics, genetics, clinical guideline development for PKU, clinical care, clinical study involvement, and dietary management.

#### Clinical-expert survey

To further explore the prevalence of PKU comorbidities and biological plausibility of the association of surrogate outcomes with comorbidity risk, a clinical expert survey was conducted. In the survey, 30 participants were identified via purposive sampling, representing similar types of experiences as those in the modified e-Delphi study, from 15 countries (Australia [*n* = 2]; Brazil [*n* = 1]; Canada [*n* = 1]; Denmark [*n* = 1]; France [*n* = 3]; Germany [*n* = 2]; Italy [*n* = 1]; Japan [*n* = 2]; Netherlands [*n* = 1]; Poland [*n* = 1]; Portugal [*n* = 1]; Slovenia [*n* = 1]; Turkey [*n* = 4]; UK [*n* = 2]; US [*n* = 7]).

### Survey design

#### Modified e-Delphi study

Survey content was informed by a targeted review of clinical literature and HTA evaluations of PKU therapies, and by qualitative discussions with multidisciplinary PKU clinical experts [2, 4, 8, 20, 24]. Seventeen distinct clinical outcomes in PKU were identified for consideration to model in CEA; these spanned the following categories: physiological/neurologic, psychiatric, psychological/behavioral, dietary/pharmacotherapeutic, maternal PKU (Table S2). In a scoping survey, eight experts rated the importance of these outcomes for CEA with regard to their impact on other PKU outcomes or comorbidities, patient/family/caregiver quality of life, patient healthcare resource use and costs, and other direct (e.g., medical foods, paid caregiving) or indirect (e.g., employment outcomes, informal caregiving) costs.

For the modified e-Delphi study, a 14-question survey was developed, addressing topics relevant to modeling the outcomes identified in the scoping survey, including association of blood Phe control with clinical outcomes, diet liberalization, unmet need, Phe-level classification, and treatment discontinuation. Given that current PKU treatments have not been evaluated in maternal PKU, questions on this outcome were not included in the modified e-Delphi survey [11–13]. Survey questions were posed as qualitative statements, and participants were asked to rate their level of agreement with each statement using a 5-point Likert scale (“Strongly Disagree”, “Disagree”, “Neutral”, “Agree”, or “Strongly Agree”). A “Don’t Know” option was also included to distinguish between neutral sentiment and indecision. A pilot test was conducted with four experts to ensure clarity of the data collection process, instructions, and questions.

**Fig. 1:**
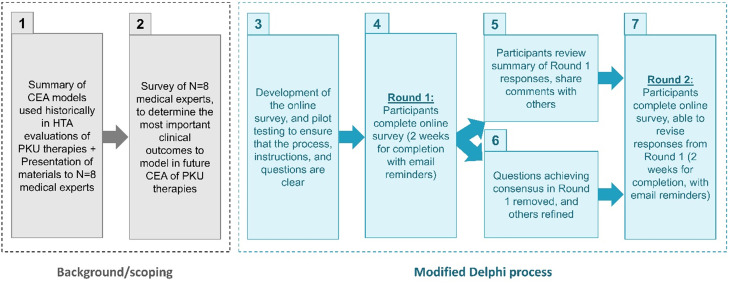
Diagram of study process. Abbreviations: CEA, cost-effectiveness analysis; HTA, health technology assessment; PKU, phenylketonuria

The modified e-Delphi study consisted of two rounds of questions and an intermediate review of participant responses and explanations, to allow participants to reassess, alter, and/or develop their opinions between rounds (Fig. 1). The responses from each of the two rounds were analyzed quantitatively to determine the degree of agreement/disagreement. Consensus was defined as ≥ 70% agreement (“Agree” or “Strongly agree”) or disagreement (“Disagree” or “Strongly disagree”), consistent with the threshold used in previous modified Delphi consensus studies [25, 26]. 

In Round 1, participants were asked to rate their level of agreement with the original 14 statements concerning key aspects of economic modeling in PKU (Table 1). For each statement rated, participants could also provide commentary on their rationale for their response. Certain questions had follow-up fields for specification of quantitative parameters based on the participant’s agreement or disagreement with the statement. Following Round 1, participants received a summary of anonymized responses for review. For each statement, participants were able to share anonymized comments via an online forum to discuss their opinions in light of the aggregated responses from their peers. Participants’ comments and specified parameter values from Round 1 were used to inform others of the group’s perspectives during the intermediate review, as well as to refine questions for Round 2.

**Table 1:** Modified Delphi study survey statements in Round 1

Survey statements (Round 1)	
Phe levels and associated outcomes	
1	An individual patient’s blood Phe levels are generally expected to be consistent within certain life stages, including (i) childhood to early adolescence (ages 0–11), (ii) adolescence (ages 12–17), and (iii) adulthood (ages ≥ 18).
2	In real-world management, a realistic (i.e., stable) measurement of a patient’s Phe level may be made in less than 6 months.
3	Phe levels of 0–29 µmol/L pose a safety risk for patients.
4	For patients with Phe levels < 120 µmol/L, Phe levels higher than 0–29 µmol/L (e.g., 30–60) may pose a safety concern for patients
5	Sustained uncontrolled Phe levels in a patient’s past are associated with intellectual disability.
6	Sustained uncontrolled Phe levels in a patient’s past are associated with outcomes other than intellectual disability.
7	Controlled vs. uncontrolled blood Phe levels are associated with near-term (e.g., within 1–4 weeks) likelihood of ADHD/executive-functioning symptoms.
Diet liberalization	
8	In real-world management, if a patient’s Phe levels reached the target range, diet liberalization (addition of dietary Phe) would be conducted only if Phe levels remained in the lower half of the target range.
9	HRQoL, if considered to include a patient’s mental health and psychosocial functioning, would be expected to improve with addition of 2–3 g of natural protein to a patient’s diet.
Unmet need	
10	The level of unmet need with the current SoC differs significantly by the 4 sapropterin-experience related subgroups listed (sapropterin naïve, failure, sub-optimally controlled, well-controlled).
11	The level of unmet need with the current SoC differs significantly by other factors (e.g., age, disease severity, pegvaliase response).
Treatment discontinuation	
12	Among patients who discontinue sapropterin treatment, negative health outcomes associated with uncontrolled PKU after discontinuation may cause patients to resume sapropterin.
13	Among patients who discontinue pegvaliase treatment, negative health outcomes associated with uncontrolled PKU after discontinuation may cause patients to resume pegvaliase.
14	Among patients who reach the target blood Phe range with pharmacotherapy, the pharmacotherapy would be continued indefinitely.

Abbreviations: ADHD, attention deficit hyperactivity disorder; HRQoL, health-related quality of life; Phe, phenylalanine; PKU, phenylketonuria; SoC, standard of care

After the initial round and the intermediate review were conducted, statements lacking consensus in Round 1, along with one statement meriting further inquiry, were refined into 10 statements for Round 2 (Table 2). In Round 2, participants were presented with the 10 statements and again rated their agreement using the 5-point Likert scale, with the option to comment on the reason for their response.

**Table 2:** Modified Delphi study survey statements in Round 2

Modified survey statements (Round 2)	
Phe levels and associated outcomes	
1	In clinical practice, most patients will generally (other than due to temporary triggers, e.g., fever) remain controlled or uncontrolled within the following age ranges: 0–4 years, 5–11 years, 12–17 years, 18–30 years, > 30 years.
4	For patients who are consuming sufficient dietary Phe (i.e., not at risk of nutritional deficiency/growth impairment), Phe levels in the range of 30–120 µmol/L may pose a safety concern.
6	The mechanisms below encompass those (both direct and indirect) that may account for the association of PKU with chronic comorbidities: high-caloric intake from Phe-restricted diet, lack of dietary protein, metabolic abnormalities associated with PKU pathophysiology, metabolic acidosis associated with medical food.
Diet liberalization	
8	If upon initiating a new therapy, a pediatric patient’s Phe levels were reduced from > 360 to consistently 240–299 for three weeks, and the patient expressed desire to consume more dietary Phe, a dietary Phe challenge may be conducted. If upon initiating a new therapy, a pediatric patient’s Phe levels were reduced from > 360 to consistently 300–359 for three weeks, and the patient expressed desire to consume more dietary Phe, a dietary Phe challenge may be conducted.
9	Medical-food consumption is reduced with increased dietary Phe (protein) consumption.
Treatment discontinuation	
12	Among patients who discontinue sapropterin treatment due to sub-optimal effectiveness (e.g., Phe levels above target range, or inability to liberalize diet), symptoms of uncontrolled PKU after discontinuation may cause patients to resume sapropterin. Among patients who discontinue sapropterin treatment due to side effects (e.g., GI discomfort), symptoms of uncontrolled PKU after discontinuation may cause patients to resume sapropterin.
13	Among patients who discontinue pegvaliase treatment due to sub-optimal effectiveness (e.g., Phe levels above target range, or inability to liberalize diet), symptoms of uncontrolled PKU after discontinuation may cause patients to resume pegvaliase. Among patients who discontinue pegvaliase treatment due to side effects (e.g., GI discomfort), symptoms of uncontrolled PKU after discontinuation may cause patients to resume pegvaliase.

Abbreviations: ADHD, attention deficit hyperactivity disorder; HRQoL, health-related quality of life; Phe, phenylalanine; PKU, phenylketonuria; SoC, standard of car

#### Clinical-expert survey

The clinical-expert survey consisted of 31 questions focusing on the association of PKU with 10 chronic comorbid conditions: anemia, asthma, chronic ischemic heart disease, depression, osteoporosis, overweight, renal insufficiency with hypertension, renal insufficiency without hypertension, type 2 diabetes mellitus, and other/unspecified diabetes. These conditions have been described in the literature as having a significantly higher prevalence among individuals with PKU compared to matched controls from the general population [5, 27–29]. 

Participants rated their agreement with statements on clinical/biological plausibility regarding: (1) whether the overall prevalence of the comorbidity is higher in individuals with PKU compared to the general population; (2) whether increased natural protein in a patient’s diet may reduce comorbidity risk; and (3) whether control of elevated blood-Phe levels may reduce comorbidity risk. The survey underwent pilot testing with seven clinical experts to appraise the clarity of the survey process, instructions, and questions.

Following pilot testing, the survey questions were administered online using the Alchemer Survey platform [16]. 

#### Validation of survey materials

During the development phase of the e-Delphi panel, the content was informed by a targeted review of cost-effectiveness modeling in health technology assessment followed by hour-long qualitative, discussions conducted with *N* = 8 international, multidisciplinary PKU medical experts to gather feedback on the study team’s interpretation of the information. From these discussions, and review of clinical guidelines and literature, 17 distinct clinical outcomes in PKU were identified across the following categories: physiological / neurologic [5], psychiatric [3], psychological / behavioral [3], dietary / pharmacotherapeutic [3], maternal PKU [3]. In the aforementioned scoping survey, the same group of experts then rated (on a 1–5 scale, limited-high) the importance of these outcomes for cost-effectiveness analysis (i.e., degree to which they affect: prognosis for other PKU outcomes / comorbidities, patient / family / caregiver quality of life, patient healthcare resource use/costs, other direct [e.g., medical foods, paid caregiving] or indirect [e.g., employment outcomes, informal caregiving] costs). The results of this scoping review (see Figure S1) were used to inform the development of the e-Delphi panel round 1 survey statements.

The content of the comorbidities risk survey was also informed by the targeted review of clinical literature and HTA evaluations, as well as the elements of the Delphi study which explored possible mechanisms for associations of PKU with comorbid conditions.

Both the e-Delphi (*n* = 4) and clinical-expert comorbidities risk (*n* = 7) surveys underwent pilot testing to ensure that the process, instructions, and questions were clear to the participants.

## Results

### A *de novo* CEA model in PKU

#### Model structure and inputs

**Fig. 2:**
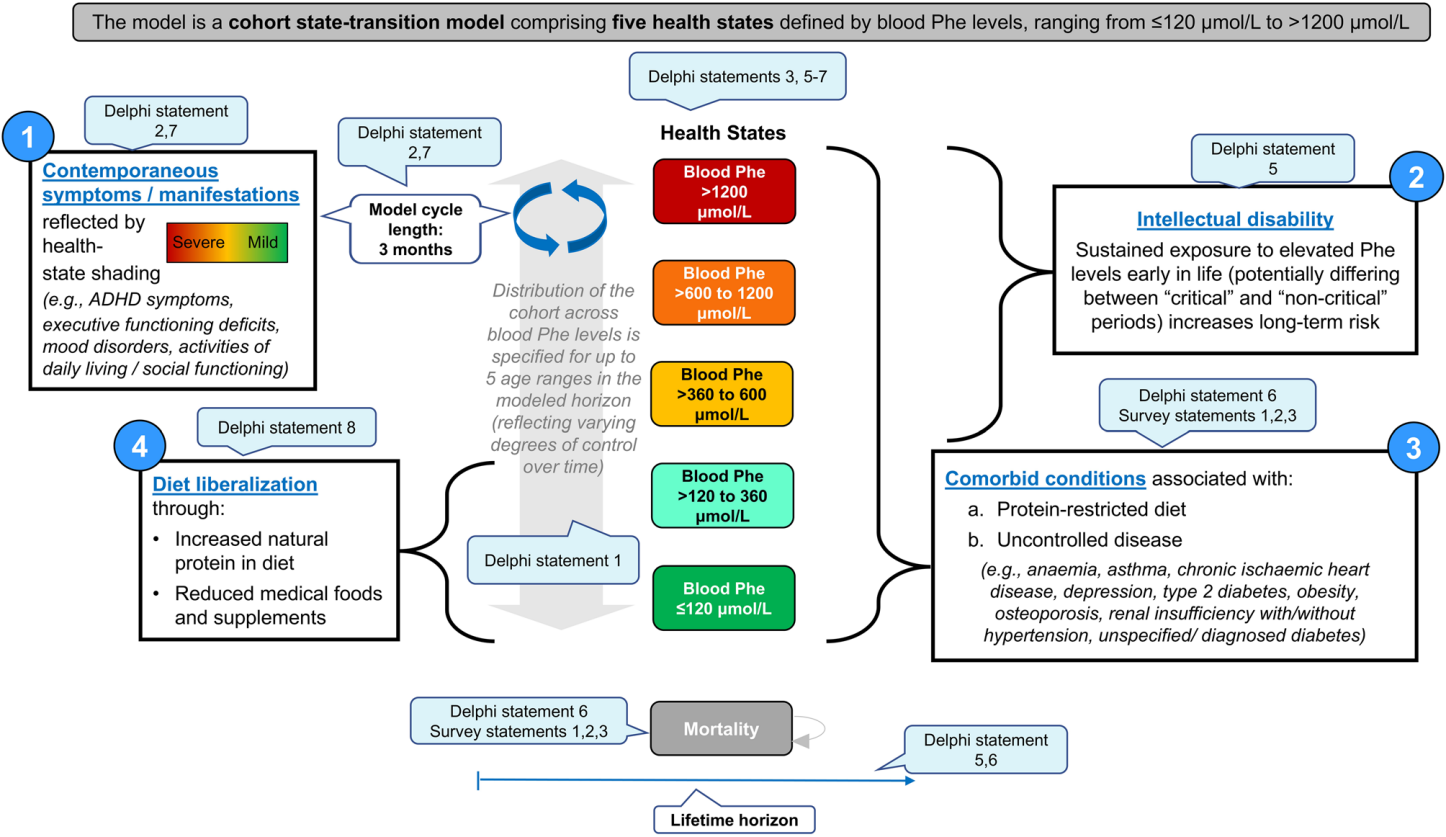
Model structure for CEA of PKU treatments. Abbreviations: ADHD, attention deficit hyperactivity disorder; CEA, cost-effectiveness analysis; Phe, phenylalanine; PKU, phenylketonuria. Note: The model, which was based on comprehensive clinical feedback, is a cohort state-transition model comprising five health states defined by blood Phe levels, ranging from ≤ 120 µmol/L to > 1200 µmol/L. Key clinical outcomes relevant to PKU burden are modeled, comprising four classes: contemporaneous symptoms/manifestations; intellectual disability; comorbidities associated with a Phe-restricted diet or uncontrolled disease; and diet liberalization achieved through either increased natural protein intake or reduced intake of medical foods and supplements

Findings from the modified e-Delphi study and clinical-expert survey informed key parameters and a conceptual structure for a de novo CEA model, as presented in Fig. 2. The contributions of the e-Delphi and survey findings to the model structure are described further below.

The model is a cohort state-transition model comprising five health states defined by blood Phe levels, ranging from ≤ 120 µmol/L to > 1200 µmol/L. This structure, in which the health states represent varying levels of blood Phe control, allows for distinguishing between health states across which associations with outcomes are reported to differ, as well as those defining management targets for blood Phe levels in clinical guidelines [4, 6]. Five-year age ranges can be specified, during which distributions of patients across blood-Phe levels can be specified for each of the treatment options compared, to reflect the consistency in control (or lack thereof) that is expected between model cycles, while allowing for changes between life periods. Comparative effectiveness of each treatment arm is thus informed by the distribution of the cohort across blood Phe levels and dietary restrictions (total daily protein-equivalent consumption, and percentage from supplemental/Phe-free foods), which influence the four classes of clinical outcomes modeled (see Section “Model clinical outcomes”).

#### Model timing

The model has a lifetime horizon and model cycle length is 3 months.

#### Model clinical outcomes

Key clinical outcomes relevant to PKU burden are modeled, comprising four classes: contemporaneous symptoms/manifestations; intellectual disability; comorbidities associated with a Phe-restricted diet or uncontrolled disease; and diet liberalization achieved through either increased natural protein intake or reduced intake of medical foods and supplements.

#### Mortality

As certain conditions comorbid with PKU have been reported to be independently associated with excess mortality risk (e.g., chronic ischemic heart disease, depression, obesity), hazard ratios for mortality can be specified by comorbid condition and applied to the background mortality rate. Excess mortality risk associated with PKU is not modeled.

### Face validity of key parameters and conceptual model structure

#### Clinical-expert survey and modified e-Delphi study results overview

The clinical-expert survey corroborated the association of PKU with increased prevalence of many chronic comorbidities (Figures S2, S3, S4). Among the comorbidities queried in the survey (anemia, asthma, chronic ischemic heart disease, depression, osteoporosis, overweight [BMI ≥ 25 kg/m2], renal insufficiency with hypertension, renal insufficiency without hypertension, type 2 diabetes, other / unspecified diabetes) a substantial percentage of participants agreed that depression (93% agreement), osteoporosis (83%), overweight (83%), type 2 diabetes mellitus (67%), anemia (60%), renal insufficiency with hypertension (53%), and renal insufficiency without hypertension (50%) were more prevalent among individuals with PKU vs. the general population.

#### Results of clinical expert survey of comorbidity risk

The majority of experts also agreed that increasing natural protein consumption reduces comorbidity risk (83% for anemia, 83% for osteoporosis, 77% for overweight, 70% for type 2 diabetes mellitus, 70% for depression, 64% for renal insufficiency with hypertension, 54% for chronic ischemic heart disease, and 52% for renal insufficiency without hypertension), a finding echoed by the modified e-Delphi study. The experts were more equivocal in their survey responses about the impact of controlling elevated blood Phe levels, but the majority agreed this would reduce the risk of overweight, osteoporosis, and depression.

#### Results of modified e-Delphi study

Among the 11 participants in the modified e-Delphi study, 100% completed the survey in Round 1, 64% contributed in the intermediate-review commentary forum before Round 2, 100% received and had the opportunity to review other participants’ responses before Round 2, and 100% completed the survey in Round 2.

##### Round 1 results

**Fig. 3:**
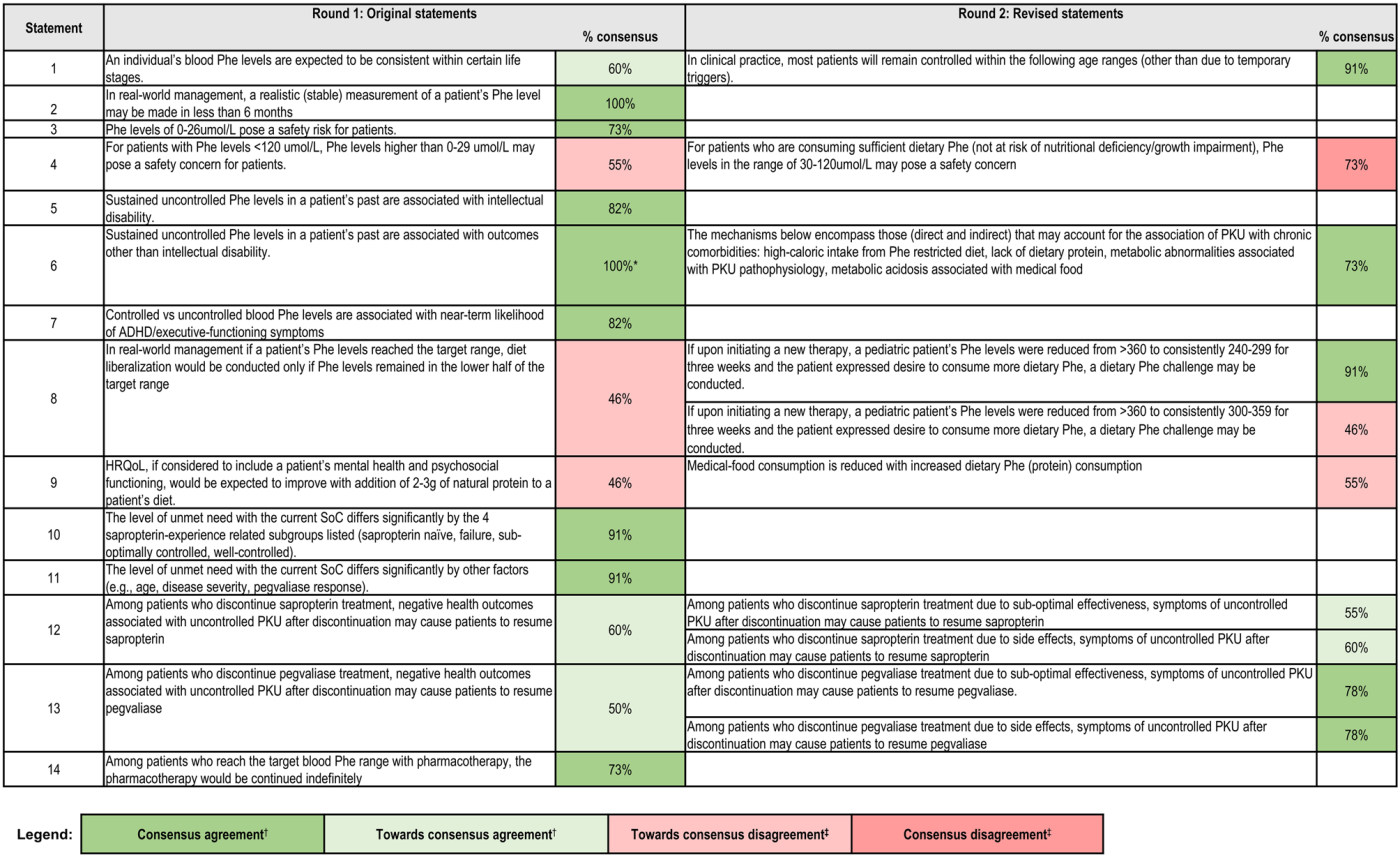
Summary of achievement of consensus in the modified e-Delphi panel, by statement and round. Note: Consensus was defined as ≥ 70% agreement or disagreement. * To inform modeling of the potential impacts of disease control throughout life on incidence of longer-term chronic conditions, it is necessary to hypothesize the mechanism(s) (both direct and indirect) for their higher prevalence in PKU. Consequently, the statement was refined to explore mechanisms for the association of PKU with chronic comorbidities, and included in Round 2. ^†^Combination of those answering agree and strongly agree. ^‡^Combination of those answering disagree and strongly disagree. Abbreviations: ADHD, attention deficit hyperactivity disorder; HRQoL, health-related quality of life; Phe, phenylalanine; PKU, phenylketonuria; SoC, standard of care

In Round 1, consensus was achieved on 8 of the 14 statements (Fig. 3). There was agreement on statements regarding the duration of time (< 6 months) required for a stable blood Phe measurement (Statement 2; 100% consensus), safety concerns at blood Phe levels of 0–29 µmol/L (Statement 3; 73% consensus), and the need to continue pharmacotherapy indefinitely among patients who reach target blood Phe levels (Statement 14; 73% consensus). There was also consensus that levels of unmet need vary across different sapropterin-experience related subgroups (Statement 10; 91%) and by other factors (Statement 11; 91%). Additionally, there was consensus that sustained uncontrolled Phe levels are associated with intellectual disability (Statement 5; 82%) and executive functioning symptoms (Statement 7; 82% consensus). Statement 6 regarding the association between sustained uncontrolled Phe levels and other health outcomes achieved 100% consensus agreement in the first round, but based on comments received in the intermediate round it was refined for Round 2.

##### Intermediate review results

In the intermediate review, a change of response by one participant led to consensus (70% agreement) following a lack of consensus (60% agreement) in Round 1 for Statement 1 on the consistency of blood Phe levels within certain life stages. For this statement, one participant changed their response from “Strongly disagree” to “Strongly agree”, due to initial misinterpretation of the question. However, because comments specified that blood Phe levels might be more consistent within different age ranges than originally stated (participants who disagreed in Round 1 specified that levels might be consistent within age ranges 0–4, 5–10, and above 25–30), the statement was refined and included in Round 2. Changes of response during the intermediate review did not lead to changes in consensus for other statements.

For Statement 4, on whether blood Phe levels between 30 and 120 umol/L pose a safety concern, comments suggested that the main concern is that patients may not be consuming enough Phe and be at risk of impaired growth, rather than pathophysiological effects of “hypo-Phe”.

For Statement 6, during the intermediate review, comments noted numerous chronic comorbidities associated with PKU, including anxiety/OCD (*n* = 6), depression (*n* = 5), tremors/hyperreflexia (*n* = 4), difficulty with social skills (*n* = 3), headache (*n* = 2), sleep disturbance (*n* = 2), school issues (*n* = 2), job issues (*n* = 2), difficulty with relationships (*n* = 2), obesity (*n* = 1), asthma (*n* = 1), bone health (*n* = 1), and ataxia (*n* = 1). Consequently, to inform modeling of the potential impacts of disease control throughout life on incidence of longer-term chronic conditions, the statement was refined to explore direct and indirect mechanisms for the association of PKU with chronic comorbidities and included in Round 2.

For Statement 8, in the intermediate review, comments generally indicated that whether diet liberalization would be conducted for blood Phe levels in the upper half of the target range would depend on the patient (e.g., their preference for dietary Phe consumption vs. blood Phe control, prior increases in blood Phe levels for addition of dietary Phe), and if so, as a “challenge” to determine tolerance on a new therapy. However, there was heterogeneity in views on how diet liberalization should be implemented. One participant expressed that diet liberalization would not be conducted individuals with blood Phe in the 301–359 umol/L range - the upper quarter of the target range - rather than upper half (per the original wording of the statement). By contrast, another participant emphasized the potential for varying blood Phe level response to dietary Phe consumption (i.e., that they do not vary hand-in-hand) by patient, describing that they had experience with “a patient in whom the increase of Phe intake did not induce the Phe level, as there is an enzymatic regulation of PAH which increased when you increase the Phe intake. This is a rare situation, but not impossible.” Based on this, the statement was amended for Round 2 into two statements delineating blood Phe levels within two ranges of the upper half of the target range.

For Statement 9, in the intermediate review, one participant changed their response from “Neutral” to “Disagree”. The comments received indicated that the amount of dietary protein associated with improved health-related quality of life (HRQoL) might be higher (e.g., 10 g) or dependent on the patient’s starting tolerance or age. Consequently, the statement was revised for Round 2 to focus on the potential to relax medical-food consumption in association with dietary Phe consumption.

For Statement 12, there was general agreement in the intermediate review that discontinuation of sapropterin is rarely due to side effects, and most commonly due to lack of efficacy. Symptoms experienced after sapropterin discontinuation which may lead to patients resuming sapropterin were also discussed. The statement was refined for Round 2 distinguishing discontinuation due to sub-optimal effectiveness vs. side effects.

For Statement 13, symptoms that may prompt patients to resume pegvaliase due to negative health outcomes after discontinuation were discussed. There was disagreement between participants on the likelihood of resuming pegvaliase if patients discontinued due to side effects. The statement was refined for Round 2 distinguishing discontinuation due to sub-optimal effectiveness vs. side effects.

##### Round 2 results

In Round 2, 10 statements were presented to the study panel (three of the statements not achieving consensus in Round 1 were refined into 6 new statements). Consensus was reached on 6 (Fig. 3). For Statement 1, similar to in Round 1, but for more specific age ranges, consensus was reached that most patients will remain controlled or uncontrolled within certain age ranges (Statement 1; 91%). For Statement 6, based on input during the intermediate round, 73% of participants agreed on direct and indirect mechanisms underlying the association between PKU and chronic comorbidities. For Statement 8, on blood-Phe levels in which diet could be liberalized, which was refined into two statements after Round 1, consensus was achieved in Round 2 on conducting a dietary Phe challenge when blood Phe levels were between 240 µmol − 299 µmol (91%) but not between the ranges of 300 µmol – 359 µmol (46%). Statement 13 on discontinuation and resumption of pegvaliase due to uncontrolled symptoms was refined in Round 2 into two statements on resumption after discontinuation due to sub-optimal efficacy and due to side effects, and both achieved 78% consensus. For Statement 4 on safety concerns for blood Phe below 120 µmol/L, there was consensus on disagreement with the statement (73%).

Some statements did not reach consensus throughout the e-Delphi process. Feedback from the HTA agencies highlighted a failure to explore the link between quality of life and the potential for diet liberalization with successful treatment in previous HTA submissions, and as such the statement “Health-related quality of life, if considered to include a patient’s mental health and psychosocial functioning, would be expected to improve with addition of 2–3 g of natural protein to a patient’s diet” was included in the initial modified e-Delphi round (Statement 9; 55% consensus). After the first round, participants commented that a more substantial increase in dietary protein intake (e.g., by 10 g or doubled) would be necessary to have a tangible impact on HRQoL. Furthermore, any detectable impact on HRQoL may be dependent on an individual’s starting tolerance or age. Additionally, they noted that what might be a perceptible improvement to a patient may not be sufficient to affect scoring on a generic HRQoL measure. One comment suggested HRQoL would be impacted by “doubling of natural protein (Phe) intake, especially if no Phe-free amino acid supplements are required.” Despite revising the statement for Round 2 to focus on the potential of increased dietary Phe consumption to relax medical-food consumption, consensus was not reached. Comments generally indicated that the ability to reduce medical-food consumption depends on the amount of the increase in dietary Phe consumption. The statement exploring the possibility of resuming sapropterin after treatment discontinuation also failed to reach the consensus threshold (Statement 12; 55–60% consensus). 

### Model structure and inputs

Development of the model structure sought to addresses HTA criticisms of prior models (Table 3). In the model (Fig. 2), inclusion of health states based on varying levels of blood Phe control was informed by the modified e-Delphi panel consensus agreement that blood Phe concentrations are associated with patient outcomes (Statements 3, 5, 6, and 7). The age ranges that can be specified were informed by consensus on Statement 1 in the modified e-Delphi panel that patients generally remained controlled or uncontrolled within the specified age ranges.

**Table 3:** Key technical and clinical criticisms raised by HTA bodies addressed in the proposed model

Agency	Issue	Model design
**Failure to capture the long-term impacts of uncontrolled Phe levels as well as long term treatment results**		
CADTH 2016 [30] NICE 2021 [31]	Long-term neurocognitive outcomes (intellectual disability / brain damage / executive functioning)	Intellectual disability is one of four key clinical outcomes classes modeled. Supported by consensus on: (Delphi statement 5)
NICE 2021 [31]	Effects on pregnant women / unborn children	Not modeled^1^
**Misalignment between health state definitions and utility/cost impacts**		
CADTH 2016 [30] PBAC 2022 [32] CADTH 2023 [33] PBAC 2018 [34]^1^	Are concurrent blood Phe levels predictive of QoL, or are other factors better predictors? Are concurrent blood Phe levels too variable for defining QoL and costs?	The model comprises five health states defined by blood Phe levels. Supported by consensus on: (Delphi statement 5) (Delphi statement 6) (Delphi statement 7)
CADTH 2023 [33]	If concurrent blood Phe levels are appropriate for health-state definition, what levels	
**Lack of support for diet liberalization modeling**		
PBAC 2018 [34] PBAC 2022 [32] CADTH 2016 [30] NICE 2021 [31] Scottish Medicines Consortium 2018 [35]	What drives QoL benefit of treatment? Blood Phe levels, or other outcomes (e.g., diet liberalization)?	Diet liberalization is one of four key clinical outcomes classes modeled.^2^ Supported by strong agreement that: (increasing natural protein consumption reduces risk of a number of comorbidities - clinical expert survey of comorbidity risk)
**Absence of modeling for PKU associated comorbidities and for caregiving impacts**		
NICE 2021 [31] PBAC 2018 [34]	Comorbidities associated with protein-restricted diet (e.g., diabetes, CVD, depression)	Comorbid conditions is one of four key clinical outcomes classes modeled. Supported by strong agreement that: (increasing natural protein consumption reduces risk of a number of comorbidities - clinical expert survey of comorbidity risk) (Delphi statement 6)

^1^Not modeled as no treatment is currently approved for use during pregnancy

^2^Consensus not achieved on eDelphi panel statement 9 “HRQoL, if considered to include a patient’s mental health and psychosocial functioning, would be expected to improve with addition of 2–3 g of natural protein to a patient’s diet”

#### Model timing

The choice of a lifetime horizon is supported by consensus agreement on Statement 5 of the modified e-Delphi panel which details the chronic comorbidities (i.e. long-term consequences) associated with PKU and aligns with criticisms of previous models with shorter term horizons being unable to capture the long-term effect of brain damage from uncontrolled blood Phe levels [31]. 

The selection of a cycle length of 3 months was informed by consensus on Statement 2, in which 100% of the panel agreed that “a stable measurement of blood Phe levels could be made in < 6 months” (the majority indicated ≤ 3 months) and on Statement 7, with 82% of the panel agreeing that “controlled vs. uncontrolled blood Phe levels are associated with near-term (e.g., within 1–4 weeks) likelihood of ADHD / executive-functioning symptoms.”

#### Model clinical outcomes

The association of blood-Phe levels with contemporaneous symptoms / manifestations is supported by clinical literature including evidence that ADHD inattentive symptoms and aspects of executive functioning have been observed to improve in patients who experienced reduced blood Phe levels [36]. Consensus agreement identified by the modified e-Delphi panel on Statement 7 regarding the association of uncontrolled blood Phe levels with near-term ADHD and executive function symptoms reinforces the inclusion of this component in the model.

Intellectual disability is incorporated as risk based on cumulative exposure (i.e., across model cycles) to elevated blood- Phe levels during early life. Published meta-analyses support the link between blood- Phe levels and intellectual disability [4, 9, 37]. Moreover, Statement 5 of the modified e-Delphi panel, “sustained uncontrolled Phe levels in a patient’s past are associated with intellectual disability” achieved 82% consensus in Round 1. The inclusion of this component addresses feedback received on prior HTAs that CEAs need to account for long-term consequences of uncontrolled blood Phe [31]. 

In patients with PKU, higher prevalence of a broad range of comorbid conditions vs. the general population has been reported in a number of studies [5, 27, 29]. In the clinical-expert survey on comorbidities in PKU, a majority of respondents agreed it is biologically plausible that risk of anemia, depression, type 2 diabetes, obesity, osteoporosis, renal insufficiency with hypertension, renal insufficiency without hypertension, and chronic ischemic heart disease may be reduced if natural protein intake is increased. The model incorporates the prevalence of these conditions as well as asthma and other / unspecified diabetes, which also are associated with PKU in published literature. Survey respondents also agreed that the prevalence of most of these conditions was higher among individuals with PKU compared to the general population and that controlling elevated blood Phe levels may reduce the risk of some comorbid conditions, including depression and overweight (Figures S2, S3, & S4). This component is further supported by consensus achieved on the modified e-Delphi panel Statement 6 that, “sustained uncontrolled Phe levels in a patient’s past are associated with outcomes other than intellectual disability.” The inclusion of the comorbidities component in the model addresses criticisms from HTA agencies on the lack of modeling the impact of comorbidities in prior models, in part attributed to lack of evidence [31, 32]. 

Diet liberalization in the model can be specified as increased natural protein intake and / or reduced intake of medical foods and supplements. The liberalization of the strict diet necessary to maintain Phe levels has been identified by patients as an outcome of particular importance and may be associated with improved HRQoL [38, 39]. Consensus on Statement 8 of the modified e-Delphi panel “If upon initiating a new therapy, a pediatric patient’s blood Phe levels were reduced from > 360 umol/L to consistently 240–299 umol/L for three weeks, and the patient expressed desire to consume more dietary Phe, a dietary Phe challenge may be conducted” further supports the inclusion of diet liberalization in the model [30–32].

#### Mortality

Mortality is modeled through the excess mortality risk independently associated with certain comorbid conditions, in line with HTA recommendations to account for the impact of comorbidity in PKU models.

## Discussion

The primary approach to PKU management involves meticulous disease management with dietary restriction of Phe intake [4, 5]. However, maintaining strict dietary control can be challenging, particularly in adolescence and adulthood [4, 7, 8]. The introduction of pharmacological therapies, such as sapropterin, pegvaliase, and the recently approved sepiapterin, has provided additional options for managing PKU, but their long-term effectiveness and cost-effectiveness remain subject to ongoing research and inquiry. Over the last decade, reimbursement evaluations for PKU therapies have underscored the lack of consensus on clinically accurate methods for health economic modeling in PKU [4]. HTA authorities have indicated that the models submitted for reimbursement evaluations have not fully addressed the clinical dimensions of the decision-problem [4]. 

Accordingly, this research was undertaken to obtain consensus and provide guidance on modeling PKU treatments and their effects on long-term outcomes. Having determined which outcomes should be modeled in a earlier scoping survey [20], expert perspectives were sought to determine a clinically informed and face validated conceptual model structure for CEA of PKU therapies using a modified e-Delphi panel (*N* = 11) and a clinical-expert survey (*N* = 30), both comprising international, multidisciplinary medical experts. The panel was chosen to reflect heterogeneity in clinical perspectives, such as those resulting from differences in European and United States clinical guidelines [4, 6]. Of the 14 statements included in the original survey for the modified e-Delphi panel, consensus was achieved for eight statements in Round 1. The remaining six statements were refined into 10 statements for Round 2, among which consensus was reached for six statements. Collectively, the entire modified e-Delphi process led to consensus statements relating to 12 of the 14 original statements.

The findings from this study provide valuable insights into the key parameters and considerations for economic modeling of PKU therapies, as well as the importance of long-term disease management and potential neurological damage resulting from elevated blood Phe levels. The experts agreed on age ranges for controlled and uncontrolled Phe levels, mechanisms linking PKU to chronic comorbidities, dietary Phe tolerance challenges, and the possibility of resuming pegvaliase after discontinuation. However, there was no consensus on whether changes to medical-food consumption could impact HRQoL or the resumption of sapropterin after treatment discontinuation. Taken together, insights from the modified e-Delphi panel and clinical-expert survey informed conceptualization of a structure for economic modeling in PKU. Importantly, the model takes into account the heterogeneity in unmet need among individuals with PKU when evaluating relative treatment benefits. The result of this work is a model with strong face validity which addresses the criticisms of previous models [15]. 

The consensus among PKU medical experts highlights the importance of clinical validation in providing guidance for economic modeling. An example of this, illustrated in this research, relates to the model cycle length. In CEA modeling, time is typically divided into discrete periods, or cycle lengths, that are selected based on the shortest expected frequency of outcomes that may change between cycles. While a previous HTA evaluation of pegvaliase stated that blood Phe levels cannot be reliably measured in less than 6 months and suggested a CEA model cycle length of ≥ 6 months, all of the participants in this study agreed that stable measurements of blood Phe levels could be made in less than 6 months [37]. Accordingly, this study suggests that a cycle length shorter than 6 months may be appropriate for CEA modeling. Other points of consensus achieved on modified e-Delphi panel statements as well as agreement identified in the clinical-expert survey also informed other aspects of the model development. The inclusion of five health states defined by blood Phe levels derives from consensus that patient outcomes, including intellectual disability and ADHD and executive functioning symptoms, are impacted by differing blood Phe levels. In addition, the modeling of clinical outcomes as contemporaneous symptoms / manifestations, intellectual disability, and comorbid conditions, are all individually supported by consensus on statements achieved by the modified e-Delphi panel.

The heterogeneity of the participants in the survey and the panel was likely of important influence in this study. With members from a range of medical disciplines across 15 countries, the expert sample in this study allowed for a broad spectrum of perspectives in PKU care to inform the model design. The heterogeneity also contributed to challenges in achieving consensus on some areas of discussion, including how best to characterize the relationship between health-related quality of life and diet liberalization. As the lack of modelling for diet liberalization has been a criticism of prior models, it has been incorporated in the present model. However, the lack of consensus on the parameters suggests the need for further research, to better quantify the impacts of diet liberalization. Points of disagreement highlight areas where future research is warranted, and for which sensitivity analysis in economic modeling may be beneficial for exploring uncertainty. For example, given the lack of consensus on the possibility of resuming sapropterin treatment following discontinuation, an option allowing for resumption could be explored in future models.

More generally, previous HTAs have indicated that important disease and treatment outcomes were omitted from economic modeling, such as the costs of long-term neurological damage resulting from uncontrolled Phe levels in childhood, the costs of potential comorbidities, the costs of caregiving, the benefits associated with lowering and stabilizing Phe levels, and the benefits associated with increased dietary protein intake for patients to achieve target Phe levels [32, 38]. Areas of consensus identified in this study on clinical outcomes of PKU and increased dietary-protein intake associated with treatment can inform clinically accurate modeling in CEA of these outcomes.

While economic models are necessarily subject to structural uncertainty, as they attempt to simplify complex disease processes and clinical management strategies, this study sought to address such uncertainty through elicitation of perspectives from a diverse panel of experts in PKU. Participants in the e-Delphi represented a range of medical disciplines supporting patients with PKU and had substantial duration of experience (all > 5 years, 10 of 11 with > 10 years, and 7 of 11 with ≥ 20 years). As such, expert perspectives received on modeling PKU are representative of multiple perspectives in PKU care. Similarly, the survey elicited clinical-expert perspectives from a diverse range of 30 experts from a range of medical disciplines with experience in management of patients with PKU across 15 countries [40–45]. While existing literature supports the higher prevalence of comorbidities in PKU in certain countries (France [27], Germany [29], and the US[5], findings from the survey help confirm existing findings more broadly geographically.

Certain limitations of this study should be noted. First, the purposive sampling used to identify the international sample of medical experts in PKU for both the e-Delphi study and the survey could introduce selection bias, limiting the generalizability of the perspectives identified. For example, epidemiological data suggest that PKU genotype severity may be highest in countries with limited representation in this research (e.g., Figure S3 of Hillert et al. [2020] indicates highest severity in Estonia, Saudi Arabia, Australia, Russia, Iran, and South Korea) [46]. However, because of the need to manage the logistical and analytical complexity within the e-Delphi process, to ensure participant engagement was facilitated, participant selection aimed to balance incorporating a broad variety of areas of expertise with geographical representation. Additionally, because the aim of the study was to elicit clinical perspectives to inform economic modeling of treatments for PKU, some of the participants recruited were identified based on their involvement in clinical studies of therapies for PKU to ensure familiarity with PKU treatments. These participants potentially had greater familiarity with the condition than other healthcare professionals. Despite these possible selection biases, it is notable that the sample, particularly for the clinical-expert survey, is sizeable considering that expertise was sought in a rare disease. Second, while the Delphi method helps to prevent an individual participant from dominating the perspectives elicited, and anonymity of participants was maintained throughout the study, the possibility of a “bandwagon effect” may remain (e.g., certain participants were more engaged in the intermediate review, which may have disproportionately promoted their perspectives with other participants) [19, 25]. Third, although pilot testing was performed to mitigate rating challenges, the broad nature of some statements may have posed rating challenges given patient-case heterogeneity encountered in clinical practice. Finally, while the present study aimed to elicit clinical expert perspectives on health economic modeling in PKU, we acknowledge the importance of incorporating patient perspectives, and this should be an area for future research.

## Conclusion

Findings from this research support the face validity of a *de-novo* economic model structure that has been developed for PKU. The modified e-Delphi study and clinical-expert survey highlight the inherent complexities in health economic modeling for PKU, attributable to the multi-system outcomes of the condition, and the intricate nature of disease management. The elicitation of clinical expert perspectives in the e-Delphi study and achievement of consensus on a number of points of uncertainty in previous economic modeling of PKU allowed for development of a clinically-informed, face-validated conceptual model structure for CEA, and identification of key accompanying parameters. In addition, the perspectives obtained from the survey are valuable for informing the economic modeling of overall burden of comorbid conditions experienced by patients with PKU and potential differences in risk of comorbid conditions with different medical-management approaches. The model developed as a result of the structured expert-elicitation approach used in this research is designed to address criticisms of earlier models, by validating blood-Phe exposures associated with other outcomes (informing health-state definitions), incorporating the long-term impacts of uncontrolled Phe levels, incorporating diet liberalization, and accounting for comorbidities associated with PKU. As such, the resulting model structure for PKU can contribute to a more comprehensive understanding of the economic implications of PKU management strategies.

## Supplementary Information

Below is the link to the electronic supplementary material.

Supplementary Material 1

## Data Availability

The datasets produced during this research are available from the corresponding author upon reasonable request.

## Abbreviations

ADHD: Attention deficit hyperactivity disorder
CEA: Cost-effectiveness analysis
HRQoL: Health-related quality of life
HTA: Health technology assessments
IQ: Intelligence quotient
ISPOR: Professional Society for Health Economics and Outcomes Research
PAH: Phenylalanine hydroxylase
Phe: Phenylalanine
PKU: Phenylketonuria
SoC: Standard of care
