# Supplementary material for: Development of a face-validated conceptual model structure for economic evaluation in phenylketonuria, through expert elicitation
Source: Orphanet J Rare Dis. 2026 May 29;21:262. doi: 10.1186/s13023-026-04423-1 (PMC13435650; doi:10.1186/s13023-026-04423-1)
Supplement: Supplementary file 1 [file 13023_2026_4423_MOESM1_ESM.pdf]

Development of a clinically validated conceptual model structure for economic evaluation in phenylketonuria, through expert elicitation

Supplementary materials

Table S1. Issues identified by health technology assessment agencies with previous PKU treatment cost-effectiveness analyses and how they were addressed in the new model

Issue	Agency	Notes from review	Findings from expert elicitation
Failure to capture the long-term impacts of uncontrolled Phe levels as well as long term treatment results			
Long-term neurocognitive outcomes (intellectual disability / brain damage / executive functioning)	CADTH 2016(36)	*"reduction in Phe levels with SAP will reduce risk of clinically significant permanent neurocognitive or neurobehavioural outcomes has not been confirmed"*	The modified Delphi study achieved consensus on the statement "Sustained uncontrolled Phe levels in a patient's past are associated with intellectual disability"
	NICE 2021(30)	"*long-term effect of brain damage from uncontrolled blood Phe levels cannot be captured in the model because of the 1-year time horizon"*	
Effects on pregnant women / unborn children	NICE 2021(30)	"*The model is not appropriate to capture the effects of PKU in pregnancy [or] for the unborn child."*	Not incorporated in model as no treatment has been approved for use during pregnancy
Misalignment between health state definitions and utility/cost impacts			
Are concurrent blood Phe levels predictive of QoL, or are other factors better predictors? Are concurrent blood Phe levels too variable for defining QoL and costs?	CADTH 2016(36)	*"neurological consequences are likely to be related to absolute Phe levels rather than relative reductions from baseline."*	The modified Delphi study achieved consensus on the statements "Sustained uncontrolled Phe levels in a patient's past are associated with intellectual disability" (Statement 5) and "Controlled vs. uncontrolled blood Phe levels are associated with near-term (e.g. 1-4 weeks) likelihood of ADHD / executive-functioning symptoms." (Statement 7) supporting the use of blood Phe levels as health states.
	PBAC 2022(33)	*"While it was biologically plausible that a lower blood-Phe was correlated with fewer neuropsychiatric symptoms in adults, the incremental benefit associated with treatment with sapropterin was difficult to accurately quantify"*	
	CADTH 2023(43)	*"The model structure does not adequately capture the condition in that a single measure of blood Phe is not sufficiently predictive of overall quality of life, blood Phe control was not related to adherence to therapy in the model, and discontinuation was not considered"* *"The Phe levels may be highly variable and can only be interpreted in conjunction with an assessment of the patient's existing diet and adherence to treatment."*	
	PBAC 2018(44)[1]	*"splitting the patient population into those with poorly-controlled and those with well-controlled blood Phe levels*	

		… was not informative because these were not two distinct subpopulations."	
If concurrent blood Phe levels are appropriate for health-state definition, what levels	CADTH 2023(43)	*"an adjustment [should be made] of health state utilities to be more in line with other chronic health conditions"*	
Lack of support for diet liberalization modeling			
What drives QoL benefit of treatment? Blood Phe levels, or other outcomes (e.g., diet liberalization)?	PBAC 2018(44)	"*These results indicate that the gain in health benefit in patients taking sapropterin to reduce blood Phe-levels is substantially greater than the gain in health benefit in patients taking sapropterin to reduce dietary restrictions"*	While the modified Delphi study was presented with the statement "HRQoL, if considered to include a patient's mental health and psychosocial functioning, would be expected to improve with addition of 2-3 g of natural protein to a patient's diet" consensus was not achieved. Diet liberalization was explored in the clinical survey in the context of comorbidity risk
	PBAC 2022(33)	*"the submission did not adequately address the range of important benefits for adults associated with lowering and stabilising Phe levels… The submission also did not address the benefit of treatment in allowing patients to increase dietary protein intake"*	
	CADTH 2016(36)	*"relationship between Phe tolerance and diet liberalization is uncertain and diet liberalization*"<br>*"All patients achieving Phe level control with SAP were assumed to have a higher quality of life than patients achieving control with diet alone; however, patients are only likely to experience improved quality of life with diet liberalization (regardless of treatment strategy)."*	
	NICE 2021(30)	*"The committee noted that sapropterin did not replace the need for diet in most people but*<br>*had been shown to be effective in combination with a protein restrictive diet (PRD) in trials. Ideally this would allow people to consume more natural protein, relax the diet to a varying degree and need less low-protein food and supplements. However, the committee concluded that the cost savings related to a reduction in protein-restricted diet are uncertain."*	
	Scottish Medicines	*"The estimated natural protein intake and reduction in the use of*	

	Consortium 2018(45)	*protein supplements and hence cost of diet in the sapropterin arm leads to significant cost offsets. There is uncertainty over the cost reduction estimated and the extent to which such cost savings can be realised in practice."*	
Absence of modeling for PKU associated comorbidities and for caregiving impacts			
Comorbidities associated with protein-restricted diet (e.g., diabetes, CVD, depression)	NICE 2021(30)	*"The committee noted that people with PKU have higher rates of comorbidities than the general population. The comorbidities seen more often in adults with PKU are: chronic ischaemic disease, urticaria, oesophageal disorders, gastroesophageal reflux disease, gastritis, anaemia, obesity, asthma, renal insufficiency, eczema, alopecia, osteoporosis, rhinitis, gall bladder disease and kidney calculus. Studies suggest that high Phe levels in people with PKU could be associated with biological mechanisms that increase the risk of chronic diseases. Also, the PKU diet, medical food and nutritional deficiencies because of the PKU diet may also contribute to the increased risk of chronic diseases. [...] The committee acknowledged that comorbidities could complicate managing the PKU diet and that caregiving for people with PKU is a burden for families, parents, and partners of people with PKU. However, it had not been presented with any evidence estimating the costs of comorbidities or caregiving, or carer disutility. It concluded that the costs of long-term brain damage after uncontrolled Phe levels in childhood, cost of potential comorbidities, and costs of caregiving and carer disutilities may be substantial and need to be considered."*	In the clinical expert survey >70% of participants agreed prevalence of comorbidities is higher in PKU vs. the general population including for depression, osteoporosis, overweight, anemia, and type two diabetes mellitus.
	PBAC 2018(44)	*"The PBAC also noted that further benefits of sapropterin therapy [were] described by patients and their families, which included a reduced risk of diabetes, reduced weight gain, improved growth and social inclusion. The PBAC considered that the consumer [patient and caregiver] input had*	

		helped define the incremental benefits of sapropterin over dietary management, in terms of meaningful patient-relevant outcomes, particularly in children and adolescents […] but considered that the [evidence submission, including CEA] had not provided sufficient evidence"	

Abbreviations: ADHD=attention deficit hyperactivity disorder; CADTH=Canadian Agency for Drugs and Technologies in Health (now Canada's Drug Agency); CEA=cost-effective analysis; CVD=cardiovascular disease; HRQoL=health related quality-of-life; NICE=National Institute for Health and Care Excellence; PBAC=Pharmaceutical Benefits Advisory Committee; Phe= phenylalanine; PKU= phenylketonuria; PRD=protein restrictive diet; QoL=quality of life; SAP=sapropterin;

[1]Australia 2018 (Pharmaceutical Benefits Advisory Committee [PBAC]); Treatment: Sapropterin; Model type: Decision tree; Horizon: 1 year, Health states: Diet status (restricted, relaxed, abandoned), Treatment (SAP vs. PRD)
[2]Canada 2016 (Canada Drug Agency [formerly Canadian Agency for Drugs and Technologies in Health (CADTH)]); Treatment: Sapropterin; Model type: Markov; Horizon: Lifetime; Health states: Adequately controlled with Phe tolerance, Adequately controlled, Not adequately controlled, Neurocognitive and neurobehavioral disorders
[3]England 2021, company; Treatment: Sapropterin; Model type: Markov; Time horizon: Lifetime; Health states: Controlled vs. uncontrolled (blood Phe >360 at ages 0-11, >600 at ages ≥12), Treatment (SAP vs. PRD)
[4]England 2021, review group; Treatment: Sapropterin; Model type: Decision tree Time horizon: 1 year; Health states: Mild symptoms + No ID, Moderate symptoms + Mild ID, Severe symptoms + Moderate ID, Treatment (SAP vs. PRD)
[5]Ireland 2017 (National Centre for Pharmacoeconomics); Treatment: Sapropterin; Model type: Markov; Horizon: Lifetime; Health state: Controlled, Partially Controlled, Uncontrolled, Asymptomatic
[6]Scotland 2018 (Scottish Medicines Consortium); Treatment: Sapropterin; Model type: Markov; Horizon: Lifetime; Lifetime: Controlled, Partially Controlled, Uncontrolled, Asymptomatic
[7]Australia 2022 (PBAC); Treatment: Sapropterin, Pegvaliase; Model type: Microsimulation; Horizon: Lifetime; Health states: Response status (blood phe <360), restricted-diet status
[8]Canada 2023 (CADTH); Treatment: Pegvaliase; Model type: Markov Horizon: Lifetime; Health states: Blood phe <120, 120-360, 360-600, 600-1200, >1200
[9]Ireland 2022 (National Centre for Pharmacoeconomics); Treatment: Pegvaliase; Model type: Markov Horizon: Lifetime; Health states: NR

Table S2. Clinical outcomes in PKU rated for importance for CEA

Category	Clinical outcome	Description
**Physiological / neurologic**	Blood Phe concentration	controlled vs. uncontrolled, or more segmented levels
	Physiological growth / development	height, weight, body mass index
	Neurologic symptoms – acute / short-term	e.g., tremors, epilepsy
	Neurologic symptoms – chronic / longer-term	e.g., dementia
	Comorbid conditions	e.g., overweight/obesity, chronic fatigue, diabetes mellitus, ischemic heart disease, dental issues, anemia, asthma, renal insufficiency, eczema, alopecia, osteoporosis
**Psychiatric**	Intellectual disability	severe reduction in IQ
	Mild-moderate reduction in IQ	
	Executive function deficit disorders	e.g., attention-deficit/hyperactivity disorder
**Psychological / behavioral**	Behavioral conditions	e.g., anger, autism spectrum disorder
	Psychological outcomes – acute / short-term	e.g., anxiety, confusion, poor mood
	Psychological outcomes – chronic / longer-term	e.g., depression, relationship satisfaction, other psychosocial manifestations
**Dietary / pharmacotherapeutic management**	Tolerance of natural Phe / protein	
	Nutritional deficiencies	e.g., deficiencies vs. recommended dietary allowances for essential vitamins and minerals
	Adverse events of treatment	e.g., hypersensitivity reactions
**Maternal PKU***	Mother's physiological health	e.g., management of morning sickness / hormone changes while on restricted diet, childbirth complications, nutritional deficiency
	Mother's psychological health	e.g., anxiety with stricter dietary control in pregnancy, concern for the unborn child
	Child's physiological health	e.g., spontaneous abortion, developmental delay and / or intellectual disability, microcephaly, congenital heart defects, low birth weight

Abbreviations: CEA, cost-effectiveness analysis; IQ, intelligence quotient; Phe, phenylalanine; PKU, phenylketonuria

*Note: As current PKU treatments have not been evaluated in maternal PKU, this clinical outcome category was not included in the e-Delphi study survey

Supplementary figures

S1: **Supplementary figures**

Figure S1: Scoping survey results -- Median (min-max) rating for importance of modeling clinical outcomes in CEA

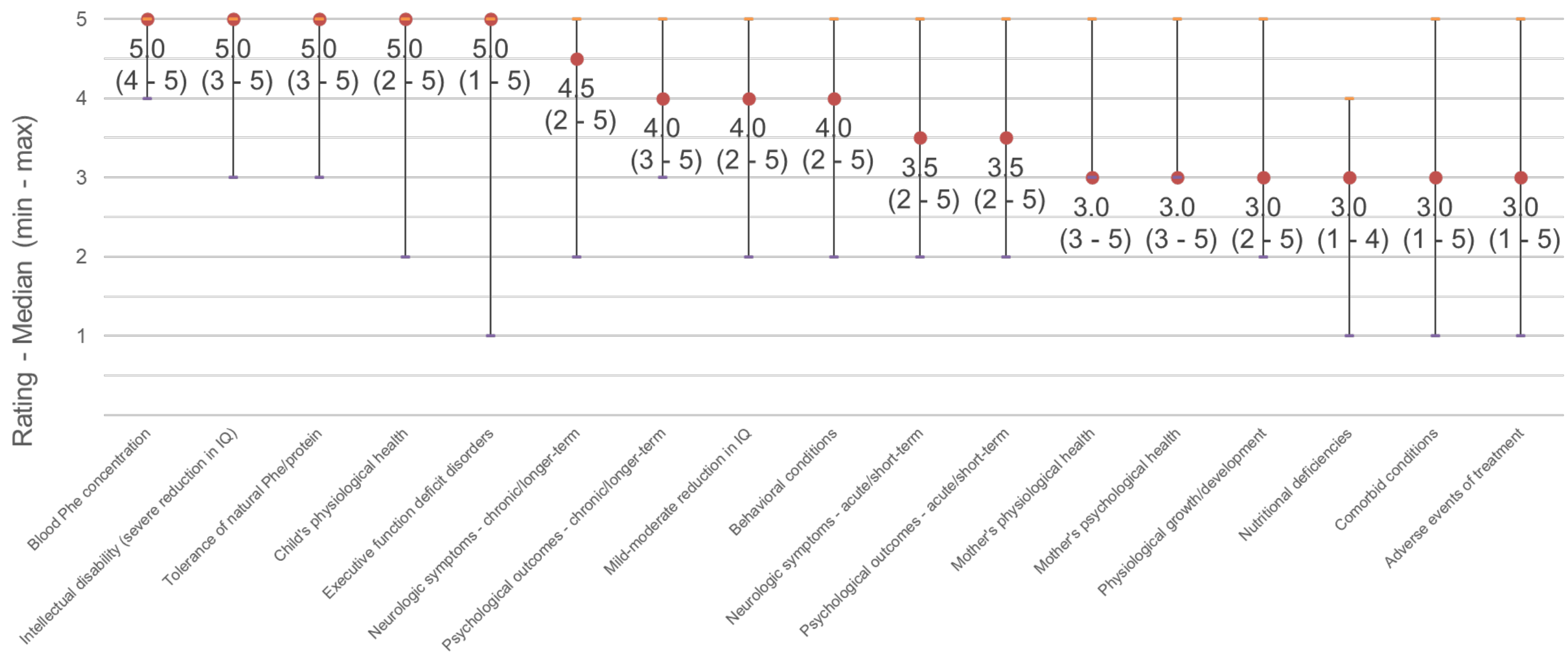

Figure S2: Clinical-expert survey - agreement with prevalence of comorbidities in PKU vs general population (N=30)

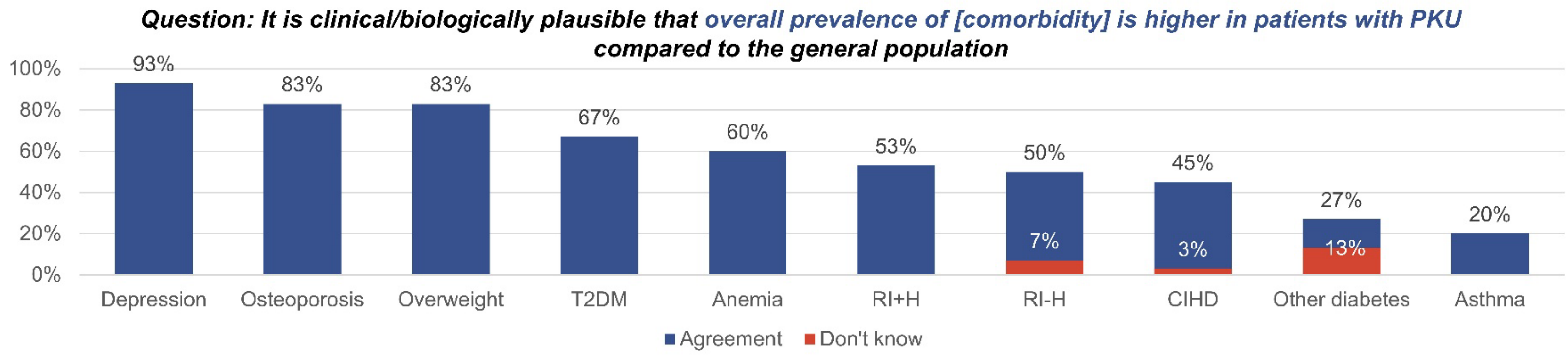

Abbreviations: CIHD, chronic ischemic heart disease; PKU, phenylketonuria; RI+H, renal insufficiency with hypertension; RI-H, renal insufficiency without hypertension; T2DM, type 2 diabetes mellitus

Figure S3: Clinical-expert survey: agreement with control of blood Phe and comorbidity risk (N=30)

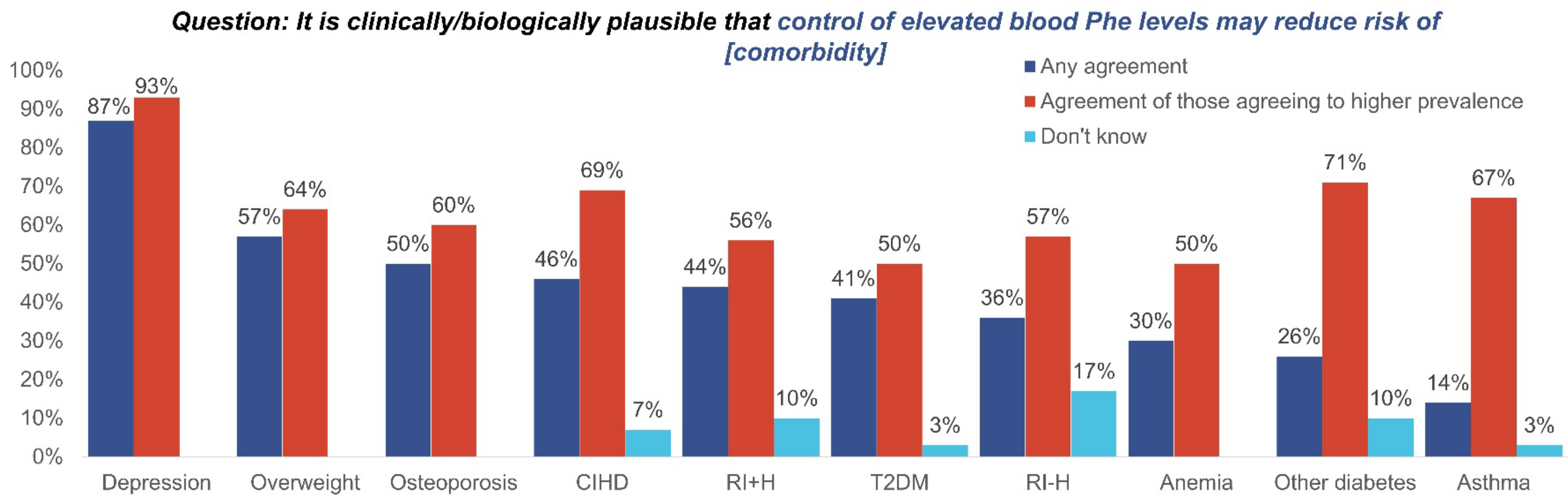

Abbreviations: CIHD, chronic ischemic heart disease; Phe, phenylalanine; RI+H, renal insufficiency with hypertension; RI-H, renal insufficiency without hypertension; T2DM, type 2 diabetes mellitus

Figure S4: Clinical-expert survey: agreement with natural protein intake and comorbidity risk (N=30)

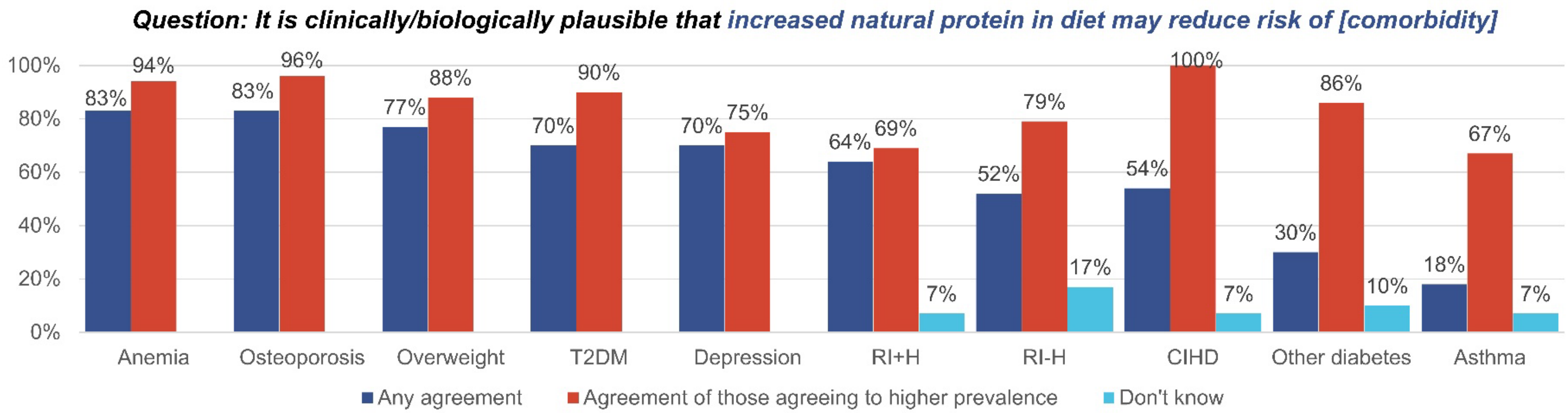

Abbreviations: CIHD, chronic ischemic heart disease; RI+H, renal insufficiency with hypertension; RI-H, renal insufficiency without hypertension; T2DM, type 2 diabetes mellitus
